# Applications of *piggyBac* Transposons for Genome Manipulation in Stem Cells

**DOI:** 10.1155/2021/3829286

**Published:** 2021-09-14

**Authors:** Yi Sun, Guang Liu, Yue Huang

**Affiliations:** ^1^State Key Laboratory of Medical Molecular Biology, Institute of Basic Medical Sciences, Chinese Academy of Medical Sciences & Peking Union Medical College, Beijing 100005, China; ^2^Department of Medical Genetics, Institute of Basic Medical Sciences, Chinese Academy of Medical Sciences & Peking Union Medical College, Beijing 100005, China

## Abstract

Transposons are mobile genetic elements in the genome. The *piggyBac* (PB) transposon system is increasingly being used for stem cell research due to its high transposition efficiency and seamless excision capacity. Over the past few decades, forward genetic screens based on PB transposons have been successfully established to identify genes associated with drug resistance and stem cell-related characteristics. Moreover, PB transposon is regarded as a promising gene therapy vector and has been used in some clinically relevant stem cells. Here, we review the recent progress on the basic biology of PB, highlight its applications in current stem cell research, and discuss its advantages and challenges.

## 1. Introduction

In 1981, Evans and Martin isolated and established separately undifferentiated embryonic stem cell (ESC) lines from the inner cell mass (ICM) of mouse blastocysts [1, 2]. Subsequently, human ESCs (hESCs) were successfully isolated by Thomson et al. [3] in 1998, and hESCs provide unparalleled tools for studying human embryonic development and regenerative medicine [4]. Additionally, mouse-induced pluripotent stem cells (miPSCs) [5] and human iPSCs (hiPSCs) were generated in 2006 and 2007, respectively [6, 7]. Two key features of ESCs and iPSCs are self-renewal, the ability to proliferate indefinitely and pluripotency and the ability to differentiate into various tissue cell types under appropriate culture conditions. As major types of pluripotent stem cells (PSCs), ESCs and iPSCs provide powerful tools to study the gene function. In particular, hiPSCs hold great promise for generating patient-specific human PSCs (hPSCs) for disease modeling and drug discoveries [8]. In addition to PSCs, other types of stem cells derived from postnatal animal tissues are widely used, such as mesenchymal stem cells (MSCs) [9], hematopoietic stem cells (HSCs) [10], and spermatogonial stem cells (SSCs) [11]. Over the past few decades, stem cell biology and mammalian functional genetics studies have developed closely together, yielding remarkable potentials for the application of regenerative medicine to drug discovery, disease modeling, and the development of novel therapeutic strategies [12].

DNA transposons are mobile genetic elements that can move throughout the genome via a “cut-and-paste” mechanism called transposition, and they are usually inactive in rodents and human cells in nature [13]. Over the past two decades, a series of active recombinant transposons have been generated and used as novel tools for functional genomics research in mice and other vertebrates [14]. Among them, *Sleeping Beauty* (SB) and *piggyBac* (PB) are the most commonly used eukaryotic DNA transposons [15, 16]. SB, a Tc1-like transposable element isolated from the salmonid fish genome, was the first transposon used in mouse and human cells [17, 18]. Although SB can function effectively in mouse somatic cells, it is not highly active in ESCs [19]. PB, however, which is derived from the cabbage looper moth *Trichoplusia ni*, shows high transposition efficiency in different mammalian cell lines, including ESCs, in addition to other organisms [20–22]. Subsequent studies have demonstrated that the translocation activity of PB is significantly higher than SB in mammalian cell lines [23]. Moreover, unlike SB, which always leaves a 2–5 bp footprint mutation after mobilization and has a strong tendency for “local hopping (reinsertion close to the original donor site),” PB exhibits the unique and valuable feature of seamless excision after transposition [24–26]. With the help of the PB system, scores of transgenic animals have been successfully generated, including mice [20, 27], rats [28, 29], pigs [30, 31], and goats [32]. Besides, PB has been used as a nonviral vector for insertional mutagenesis [33], genetic screens [34–38], iPSCs engineering [39–41], gene therapies [15, 42–45], and novel CAR-T cell therapeutic strategies [46–49]. In this review, we will look back to the advancements of PB transposon in stem cells and regenerative medicine, and discuss its wide applications, so as to provide a reference for future research.

## 2. Characteristics of the PB Transposon

### 2.1. Integration Site Preference

**Figure 1:**
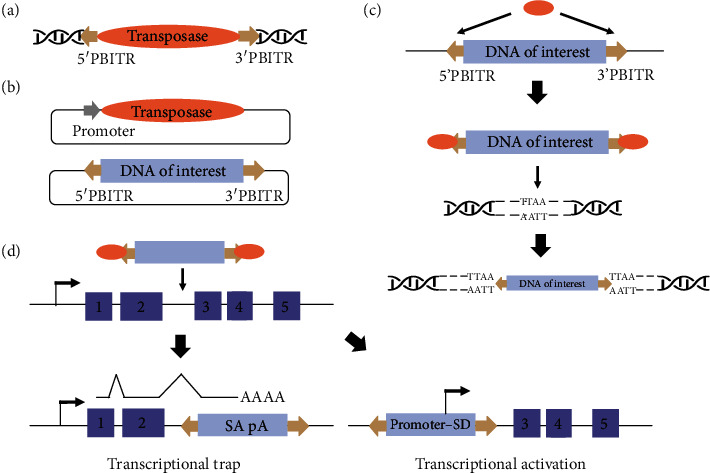
The *piggyBac* (*PB*) transposon System. (a) The original PB elements consist of the transposase gene (orange) and inverted terminal repeats (ITRs, yellow arrows). (b) A binary transposition system for gene delivery in plasmids. One is a transposase expression helper plasmid driven by a promoter (grey arrow), and the other is a donor plasmid that contains a DNA sequence of interest (light purple) and/or drug selection marker flanked by ITRs. (c) Vector-to-chromosome transposition. After cotransfection of the binary system in vitro or in vivo, the transposon carrying a transgene is excised from the donor plasmid and then integrated into the chromosome via transposase interaction with host genome sites containing TTAA segments. (d) Insertion of transposons can disrupt or promote gene expression. In the example shown in this figure, a transposon is integrated between exons 2 and 3 (numbered purple), which may lead to two possible outcomes: (1) the transposon hijacks transcription via the splicing receptor-polyadenylate signaling (SA-ployA) element, disrupting gene function and leading to the trap of transcript expression (exons 1–2), and (2) the transposon drives the expression of downstream gene sequences (exons 3–5) via the promoter-splicing donor (SD) element.

The PB element was originally discovered in insect cells as a repetitive element while propagating baculovirus in the TN-386 cell line as shown by Fraser et al. [50] and isolated by Cary *et al*. in 1989 [51]. The inserted mobile DNA was carried by the virus in the form of a “piggyBack,” hence the name *piggyBac*; “*Bac*” stands for it being a baculovirus-related discovery. In 2005, Ding et al. found that PB elements can actively transpose in a variety of human and mouse cell lines, as well as in mouse germline cells [20]. The original PB element is a 2,475 bp fragment within an open reading frame (ORF) that encodes a functional transposase of 594 amino acids, flanked by 311 bp 5′ end and 235 bp 3′ end sequences, each containing asymmetric inverted terminal repeats (ITRs) carrying transposase binding sites (Figure 1(a)). The 35 bp 5′ end ITR (5′PBITR) and 63 bp 3′ end ITR (3′PBITR) were shown to be sufficient for activity both in vivo and in vitro [52]. Importantly, the PB element can be divided into two functional components, ITRs and the PB transposase (PBase), to form a binary transposition system, and have been split into a helper plasmid and a donor plasmid (Figure 1(b)). The PBase, which can be transiently expressed by the helper plasmid, excises any DNA sequence of interest flanked by the ITRs in circular donor plasmid via binding to the ITRs (i.e., cut) and reintegrates the sequence into the TTAA site in the genome (i.e., paste) (Figure 1(c)) [53, 54]. The insertion site can be detected using Splinkerette PCR combined with DNA sequencing [34, 55]. The further advantage is seamless excision that the reexpression of PBase can remove the transposon completely to obtain transposon-free cells [56].

Several studies have shown that the distribution of PB transposons has no correlation with gene density or expression level, but rather depends on the distribution pattern of TTAA sites [57] and was negatively influenced by genomic methylation [22, 58]. Theoretically, there is an average of one TTAA site every 256 bp (four to the power of four) in the genome, but the protein-coding regions have a higher GC content compared to other positions, leading to a lower frequency of TTAA sites [59]. In addition, only about 1% of PB insertion sites are located in the 5′ region within 1000 bp upstream of the transcription start site (TSS), which is much lower than the proportion for retrovirus systems [22, 58, 60]. Compared to lentivirus systems [21, 60], PB preferably integrates into genomic safe harbors (GSHs), which are defined based on five criteria for its relative location to ultra-conserved regions, noncoding RNAs, and coding genes, especially cancer-related genes [61, 62].

### 2.2. Mutagenic Cassettes

As discussed above, transposons acted as DNA delivery vehicles for genetic modifications. Several PB-based vectors that have been used for insertional mutagenesis contain two main features. These are (1) mutagenic gene trap cassettes to mediate target gene expression (loss or gain-of-function, LOF, or GOF) and (2) reporter cassettes, whose expression is dependent or independent of the correct splicing between exons of the trapped gene and mutagenic gene trap cassettes [63, 64]. Based on the strategy used for mutating genes, gene trapping can be mainly divided into promoter trapping and polyadenylation (polyA) trapping [65]. In promoter trapping, mutagenic cassettes usually include a splice acceptor (SA) followed by reporter genes and polyA signals in one or both orientations. After integrated into an intron of the expressed gene, the SA-report-polyA element can disrupt the expression of the trapped gene by splicing into upstream exons, which results in a gene trap fusion transcript, and the expression of reporter gene is driven by the endogenous promoter of the trapped gene. As the expression of such a reporter cassette depends on an endogenous promoter, they can only drive transcriptional activation in a tissue of interest [63]. Thus, a reporter driven by an exogenous promoter can be separately used and is independent of the splicing fused transcript, which has allowed more than 90% of mutational coverage of all mouse genes with unbiased distribution throughout the genome [66]. The reporter cassettes used are usually fluorescent proteins (e.g., green fluorescent protein, GFP; red fluorescent protein, and RFP), antibiotic resistance (puromycin, neomycin, hygromycin, etc.), or *β*-galactosidase.

In polyA trapping, transposon insertions utilize a unidirectional exogenous strong promoter followed with a splice donor (SD), but lacks a polyA signal (Figure 1(d)). If the orientation of the exogenous promoter-SD element is consistent with the direction of the transcription of the trapped gene, the element will be spliced into endogenous, downstream exons, hence initiating gene transcription regardless of transcriptional activity [63]. Some trap cassettes with strong viral enhancers/promoters may result in overexpression of truncated or full-length protein products of the trapped gene. Moreover, the promoter of the trapped gene may be transactivated by strong enhancer elements inside the transposon, leading to the overexpression of a full-length transcript [63]. It is worth noting that vector integrations always tend to occur in the last introns (3′-end most) of the trapped gene in poly-A trapping. By inserting an internal ribosome entry site (IRES) sequence between the reporter cassette and the SD site to prevent nonsense-mediated mRNA decay (NMD) of chimeric transcripts, the bias in the vector integration site can be effectively removed [67]. These features enable the PB system to be a rapid, high-throughput, and traceable mutagenesis tool for constructing mutant libraries for LOF or GOF screening and identification of insertional genes for further validation.

### 2.3. Cargo Capacity

Genomic sequences contain protein-coding regions and important *cis*-acting regulatory elements (promoters, enhancers, repressors, etc.) that are essential for appropriate spatial–temporal gene expression. Therefore, the capacity to deliver large cargo is critical for achieving successful gene expression regulation. Retroviral and lentiviral vectors' cargo capacity is restricted to about 10 kb and also has immunogenic and tumorigenic potential [68]. Nonviral systems, such as SB transposon, are also limited to 5–6 kb in cargo size and have shown a reduced transposition efficiency when cargo size reaches 10 kb [69]. These characteristics limit the use of selectable markers, inducible cassettes, and large regulatory sequences. However, Li et al. showed that in mESCs, giant PB transposons could mobilize 100 kb DNA fragments to endogenous genomic sites with good cargo integrity, and transposons could be seamlessly excised after transposition [70]. Since the transposition efficiency decreases with increasing cargo size, 100 kb is unlikely to be the upper limit of PB cargo capacity. In general, PB can carry multiple genes during transposition, providing great advantages for multiplexed genetic manipulations, including insertional mutagenesis.

### 2.4. Transposase

Engineering the PBase is the key to enhancing PB transposition efficiency in mammalian cells. A mouse codon-optimized version of the PBase (mPBase) mediates a 20-fold increase in vector-to-chromosome transposition relative to the original native version [54] and also elevated the rates of chromosomal transposition from PB donor loci in mESCs [71]. The enhanced *PiggyBac* (ePiggyBac) system, which contains a human codon-optimized transposase and the T53C/C136T mutant 5′PBITR, could increase genome integration efficiency by 10-fold in hESCs [72]. Subsequently, a hyperactive PBase (hyPBase), with a total of 7 amino acids (aa) substitutions as shown by Yusa et al., can mediate more efficient transposition and outperformed the mPBase by 10-fold without compromising genomic integrity [73]. An in vivo study reported that the hyPBase had a 20-fold increase in the liver-directed expression compared to mPBase [74]. Moreover, it is well known that the PB transposon can be excised by the reexpression of transposase, but there is still the possibility of transposon jumping into new locations. To solve this problem, Li et al. generated an excision competent/integration defective (Exc^+^/Int^−^) PBase by amino acids mutation at a catalytic domain [75]. As the integration of the PB transposase vector into the host genome may lead to multiple transposition cycles, scientists have discovered that transfection of PB transposase mRNA (a short half-life) instead of a plasmid can effectively reduce the potential genetic toxicity [76, 77]. An optimized PB transposon system will significantly expand its application in various fields.

### 2.5. Comparison/Combination with Other Nucleases

The PBase is a very efficient enzyme that actively integrates DNA fragments into the genome in a random manner [78]. Recently, engineered nucleases, including transcription activator-like effector nucleases (TALENs), zinc finger nucleases (ZFNs), and clustered regularly interspaced short palindromic repeats (CRISPR)/CRISPR-associated 9 (Cas9), have been widely used for gene transfer and modification through generating double-strand DNA breaks (DSBs), which can be repaired by homologous directed recombination (HDR) [79]. However, all of these systems exhibit offtarget effects and nonenzymatic DNA insertion [80]. Thus, some works have sought to design PBase fused with these nucleases for integrating DNA into a unique user-defined chromosome site. Although the chimeric TALE-PBase [81] or ZFP-PBase [82] targeting of a unique genomic locus increased transposition efficiency, no targeted transposition was demonstrated [83]. The CRISPR/Cas9 system uses a short guide RNA (sgRNA) to guide the DNA endonuclease Cas9 to a specific target site and facilitates mutation insertion [84]. A specific Cas9 mutant lacking endonuclease activity (dCas9) fused with transcriptional repressor or activation domains has also been generated to promote transcriptional inhibition or activation when coexpressed with targeted sgRNAs [85, 86]. Lena et al. fused dCas9 to PBase and targeted it to specific genomic sites using dual sgRNAs [87]. Thus, the ease of design and application of dCas9-PBase, which can edit genes at precise genomic loci, improves future medical applications.

## 3. Functional Genomics Using PB-Mediated Genetic Approaches

Identifying genes that are important for specific biological phenotypes and diseases is a crucial goal of genetic analysis, and genetic screens have proven to be one of the most effective approaches [88]. The reverse genetic analyses are hypothesis-driven investigations of a phenotype driven by the disruption of predefined genes [89], while forward genetic screens are phenotype-derived approaches that generally involve high-throughput mutant libraries generation, specific phenotype selection, and mutations validation [90].

### 3.1. Loss-of-Function Screening

LOF genetic screens using mammalian cell lines are powerful tools for identifying genes required for many cellular processes. Since ESCs can differentiate into a variety of cell types, including germ cells, and have relatively stable genomes amenable to various genetic manipulations, these cells have become attractive models for analyzing developmental events or disease phenotypes in vitro [80]. With the excellent mutagenic ability of PB transposon and less bias towards certain genomic hot spots, a genome-wide mutant library could be rapidly and efficiently established in ESCs, allowing researchers to perform phenotype-based genetic screening in mammalian cells, similar to studies that have been done in yeast for the past 30 years [91]. In combination with high-throughput next-generation sequencing (NGS) technologies [91, 92], hundreds to thousands of genes trapped by PB transposons could be easily identified, enabling the study of the molecular mechanisms of practically any biological process studied (Table 1).

#### 3.1.1. The Problem of Homozygosity Mutations

Since most of phenotypic changes in mammalian cells require both copies of an autosomal gene to be inactivated (except in some cases of haploinsufficiency) [93], the genome-wide LOF screen of recessive mutations is quite time-consuming and rather difficult using diploid cells [94]. This issue was partially solved by generating a Bloom's syndrome gene- (*Blm*-) deficient ESCs, which lead to a higher rate of mitotic recombination between sister chromatids. *Blm*-null ESCs harboring heterozygous mutation conversed to homozygous mutations through a loss-of-heterozygosity (LOH), which occurs at a rate of about 10^−4^ events/locus/cell/division. Thus, a mutant library needs to be expanded for at least 14 population doublings to promote homozygous mutant generation for further LOF screens, such as the resistance to 6-thioguanine (mismatch repair mutants) and retroviral infection [34, 95–97]. Huang et al. used a PB transposon vector, which carried two drug resistance genes but could express only one at a time and *Blm*-null ESCs to isolate homozygous mutant cell clones successfully. The two expressed drug-resistant genes could be switched by Cre recombinase, and this allowed selection for the increase in homozygous mutants that occur after LOH [94]. However, due to the low frequency of LOH in each generation per cell and homozygous cells accounting for only a very small fraction of *Blm-*null cells, it is not easy to achieve a sufficient number of homozygous mutants for genetic screening [91]. Recently, as an encouraging breakthrough in cell biology, haploid ESC (haESC) lines have been generated in several species, including medaka fish [98], mice [99–102], rats [103], monkeys [104], and humans [105–107]. As there is only one set of chromosomes in haploid cells, it becomes quite easy to generate loss-of-function mutations using haESCs, which hold great promise for both forward and reverse genetic screens [38, 91, 99, 108–116].

#### 3.1.2. Stem Cell Characteristic-Related Screening

Due to the infinite self-renewal ability and haploid properties, haESCs have become powerful tools for generating a tremendous number of homozygous mutation pools [117]. The PB transposon system has also been successfully applied to haESCs to identify different mechanisms of stemness and differentiation. Although the mechanisms of self-renewal of PSCs have become clearer, less is known about how these robust pluripotency programs are modulated to enable fate transitions. PB-mediated large-scale libraries in haESCs for the genetic exploration of the exit-from-pluripotency have been reported, and researchers have identified the RNA binding protein Pum1 and the conserved small zinc finger protein Zfp706 as being required for exit from self-renewal state timely and efficiently [108]. In addition, the combination of PB with newly established haploid stem cell lines from other cell types also plays a vital role in the study of lineage-specific functional genomics. Recently, Cui et al. generated mouse parthenogenetic haploid trophoblast stem cells (haTSCs), which can also serve as a powerful tool for forward genetic screens in placental biology and disorders [117]. In another study, Peng et al. obtained haploid-induced trophoblast stem cells (haiTSCs) from *p53*-deficient haESCs by overexpressing the *Cdx2* gene in vitro. PB-mediated high-throughput mutation in haiTSCs was performed and used to screen factors related to the trophoblast lineage, and then *Htra1* was validated as a blocker of spongiotrophoblast specification [118].

Mouse epiblast stem cells (EpiSCs) are derived from the postimplantation egg cylinder epiblast. Unlike ESCs in a naïve pluripotent state, EpiSCs are in a primed pluripotent state and have been widely used to explore the intricate mechanisms of reprogramming [119–121]. Recently, Gao et al. established haploid EpiSCs (haEpiSCs) from mouse postimplantation epiblast at embryonic day 6.5 (E6.5) by microinjecting *p53*-knockout haESCs into normal blastocysts. Through a massive PB-mediated mutagenesis protocol, researchers determined *Hs3st3b1* as a key modulator that may impede the reprogramming process, providing a valuable resource for reprogramming research [122].

Although haploid stem cells have many advantages in genetic screening, the haploid state is generally unstable in culture. As haESCs tend to become diploids spontaneously, it hampers their application in functional genomic researches [99, 100]. In a recent study, we used a genome-wide haESC homozygous mutant library based on PB transposon mutagenesis to screen the potential haploidy-maintenance factors and found that *Etl4*-deficiency reduced the rate of self-diploidization in haESCs. This gene was found to be linked to an energy metabolism transition, thus providing a novel strategy for maintaining haploid status during cell culture by regulating cell metabolism [123].

#### 3.1.3. Drug Resistance Screening

Pettitt et al. used a PB transposon-based dual-directional gene trap vector and mouse haESCs to generate large-scale gene mutant libraries. The resistance to olaparib, a clinical poly (ADP-ribose) polymerase (PARP) inhibitor, was screened, and it was determined that the toxicity of olaparib in normal cells was mainly mediated by PARP1 [124]. At present, almost all reported genetic screens based on mixed mutant pools must rely on strong positive selections of resistant clones, and “negative selection”-based screens are not easy to conduct using these mixed pools due to the possible interference and interplay among different mutant cells, which can interfere with the readout of the quantitative deep sequencing of such a screen [125]. Therefore, we generated arrayed haploid mutant libraries with up to 85% homozygous mutant clones and then conducted a negative screen to discover mutations conferring sensitivity to the DNA-damaging drug doxorubicin, an anticancer drug frequently used in clinic [91]. Recently, Mao et al. developed an inducible self-inactivating PB system (named “One-Shot”) that allows rapid construction of a mutant library in mouse haESCs and haploid neural stem cell-like cells (haNSCLCs) with single-copy mutation site per cell and puromycin-related resistance was chosen to evaluate this system [126]. Through PB transposons, high-throughput trap mutations can be effectively integrated into haploid neural progenitor cells (haNPCs), which can remain haploid and maintain the potential to differentiate into neurons and glia for long periods in vitro. The target genes of a tetrodotoxin-like toxicant A803467 (B4GALT6) were uncovered subsequently using such a strategy [38]. These studies have expanded the scope of genetic screens in mammalian cells.

### 3.2. Gain-of-Function Screening

In addition to LOH screens, forward genetic analysis using PB-based GOF mutagenesis enables researchers to more fully explore various biological processes functionally. Since the genetic changes acquired during the culture of hPSCs may influence their availability for research and future treatments, Weissbein et al. used a PB transposon vector that contained the cytomegalovirus (CMV) enhancer and promoter sequences followed by the SD from the rabbit beta-globin intron, to construct genome-wide libraries of hPSCs. After screening, they uncovered that the overexpression of the RAS pathway led to resistance to the hPSC-specific drug PluriSIn-1, and inactivation of the RHO-ROCK pathway resulted in a growth advantage in culture adaptation [127].

As discussed earlier, EpiSCs may be the barrier in somatic cell reprogramming. Therefore, Guo et al. performed a genome-wide PB insertional activation screen in EpiSCs to identify the factors that can overcome the impediment between EpiSCs and iPSCs [35]. The gene-trap activation vector contained a murine stem cell virus (MSCV) long terminal repeat (LTR) with an SD site from exon 1 of mouse *Foxf2*, which could promote full or truncated protein expression when integrated upstream or within a gene [128]. To date, GOF screening using transposons has been relatively rare, and this has usually been in combination with a LOF to form a bifunctional activating and inactivating transposon system. For example, transgenic mice with these bifunctional activating and inactivating transposons, which carry different promoter/enhancer elements and bidirectional SA with SV40 polyA signals, have been used for the discovery of oncogenes and tumor suppressor genes [27].

### 3.3. Comparison with Other Screening Systems

Other forward genetic screening methods in functional genomics research include cDNA libraries, RNA interference (RNAi) libraries, and libraries using the CRISPR/Cas9 system for GOF or LOF screens [129]. Compared with transposon-induced mutagenesis, these methods each have distinct advantages and disadvantages, and the combination of different methods can provide complementary techniques for uncovering functional genes (Table 2).

## 4. PB in Stem Cell-Based Preclinical Studies

Stem cells, as ideal targets for gene therapy, require effective tools for the transient or permanent transfer of genetic information into eukaryotic genomes. Through transposon-based genetic manipulation, therapeutic genes can be introduced with stable phenotypic correction, and stem cells edited can be expanded in vitro, followed by differentiation into particular cell lineages for specific therapeutic needs. Currently, there is widespread evidence that robust transposon-mediated gene transfer can be achieved in several clinically relevant stem cell types, such as hESCs, iPSCs, HSCs, MSCs, or myoblasts.

### 4.1. hPSCs

Over the past two decades, culture conditions have been a major focus for hPSC research [3, 130]. Recently, extended or expanded pluripotent stem cells (EPSCs) have been reported to have the additional ability to contribute to both embryonic and extraembryonic tissues [131–133]. It was pointed out that Gao et al. generated doxycycline- (Dox-) dependent porcine iPSCs via stable genomic integration of complementary DNA (Yamanaka factors *OCT4, MYC, SOX2*, and *KLF4* together with *LIN28, NANOG, LRH1*, and *RARG*) in porcine fetal fibroblasts (PFFs) using PB transposition. Under similar conditions, hESCs and hiPSCs can be transformed into EPSCs [132, 134]. The successful generation of EPSCs provides tools for embryogenesis and transformation research in regenerative medicine. In addition, hPSCs can also be used for the construction of disease models and therapeutic applications. COVID-19, caused by severe acute respiratory syndrome coronavirus 2 (SARS-CoV-2), has been declared a global pandemic by the World Health Organization. In addition to respiratory failure, COVID-19 can cause clinical complications in other systems, including metabolism, the heart, the nervous system, and the gastrointestinal tract [135]. An hESC line WAe001-A-58 was generated by PB transposon vector, which carried the Tet-On gene expression system of the SARS-CoV-2 nucleocapsid (N) protein-coding sequence, from the hESC line WA01 (H1), providing an ideal platform for further elucidating the pathological role of the N protein [136].

### 4.2. iPSCs

Initially, Dr. Shinya Yamanaka and his colleagues expressed four genes (encoding transcription factors Oct4, Sox2, Klf4, and c-Myc) in somatic cells using retroviruses, and these somatic cells were reprogrammed into an embryonic-like state with similar developmental capabilities [5]. However, due to safety concerns, permanent insertion of the virus in the genome may limit the clinical applications of iPSCs. Using the PB transposon system, mouse and human iPSCs have been successfully generated, and reprogramming factors can be removed from these pluripotent cells without any traces via the reexpression of PBase [137, 138], thus minimizing potential concerns associated with insertional oncogenesis. To better control copy numbers in the genome, all four reprogramming factors can be introduced into one vector using approximately 20 amino acid long self-cleaving 2A peptides to separate these different genes [137, 138].

In recent years, PB transposons, combined with TALENs or the CRISPR/Cas9 system, have been used for the genome editing of iPSCs to correct gene defects [25, 26, 139, 140]. Genome editing relies on the introduction of double-strand breaks at target sites using “nucleases” to allow the occurrences of error-prone nonhomologous end-joining (NHEJ) or HDR near the nuclease cutting site, followed by the traceless removal of selectable gene fragments via PBase [24]. This strategy has recently been used to achieve the correction of mutations in the hemoglobin beta chain gene. By combining PB with TALENs or CRISPR/Cas9, the mutated *β*-globin gene in sickle cell disease- (SCD-) specific iPSCs or *β*-thalassemia patient-derived iPSCs was successfully seamlessly corrected without any detectible offtarget or adverse chromosomal alterations [25, 26]. Similarly, it has recently been shown in iPSCs derived from patients with Huntington's disease that the combination of PB transposon with the CRISPR/Cas9 system may support gene therapy in these genetic disorders induced by trinucleotide repeat expansion [141]. Corrected stem cells successfully differentiated into excitable, synaptically active forebrain neurons.

Genetic manipulation of iPSCs before transplantation may further threaten genomic stability, which can affect their differentiation, characterization, tumorigenicity, and uncontrolled cellular behavior [142]. Therefore, whole-genome sequencing is needed to detect such changes [143], and more preclinical trials in mice and other animal models will be necessary to further confirm the therapeutic potential of reprogrammed cells in vivo [144].

### 4.3. HSCs

HSCs are ideal tools for gene therapy in hematologic diseases due to their ability for self-renew and differentiation into different lymphohematopoietic lineages. The PB transposon system has been used for stable gene transfer of CD34+ HSCs; although, comparative analysis has shown higher activity of SB100X, the most hyperactive version of the SB transposase currently [145, 146]. PB transposon-modified HSCs continue to express functional globin chain proteins, exhibiting a reduced sickle phenotype and an improvement in disease progression. Later, the hyPBase, which is more active than SB100X in other cell types, has been developed [147], but the comparison with SB100X in HSCs has not been performed yet.

### 4.4. Mesenchymal/Stromal Stem Cells

Human MSCs originate from human embryonic mesoderm and/or can be isolated from fetal and adult tissues, such as bone marrow (BM), umbilical cord (UC), adipose, etc. [9], and are a heterogeneous subset of nonhematopoietic multipotent stromal stem cells. MSCs can be differentiated into ectodermal (e.g., neuronal cells), mesodermal (e.g., osteocytes, chondrocytes and adipocytes), and endodermal lineages (e.g., hepatocytes). It has been reported that the PB system was applicable to gene integration in MSCs [148]. Yang et al. generated immortalized human UC-derived MSCs (iUC-MSCs) using the PB-based monkey virus 40 T antigen (SV40T) system. These cells positively expressed MSC markers and did not induce tumorigenesis in vivo with the retained potential for trilineage differentiation after BMP9 stimulation, which has laid a foundation for further study and applications in UC-MSCs [149]. Moreover, MSCs are considered excellent cancer therapeutic tools in view of their unique ability to target tumor cells. Interferon-gamma- (IFN-*γ-*) expressing adipose-derived MSCs (AD-MSCs) generated by PB-mediated gene transfer were engrafted into tumor stroma in a mouse model of melanoma and could inhibit tumor growth and angiogenesis, prolong the survival of mice and exhibit an important implication for future cancer treatment [150].

### 4.5. Myoblasts

Myoblasts are self-renewing adult muscle progenitor cells that can eventually differentiate into skeletal muscle fibers for the potential treatment of muscle disorders. Reports have shown that PB-mediated gene transfer can be used to deliver therapeutic genes into myoblasts effectively. Based on the use of the PB transposon system, the genes encoding either full-length human dystrophin or truncated microdystrophins could be successfully introduced into myoblasts and expressed in differentiated multinucleated myotubules [151], paving the way toward a PB-mediated gene therapy approach for Duchenne muscular dystrophy (DMD).

### 4.6. Safety Issues of *piggyBac*

DNA transposons allow nonviral stable gene transfer and potentially replace the need for viral vectors, but there are still a few safety issues to consider carefully.

- Insertional mutagenesis is one of the major concerns of any integration-based gene therapy. Since PB transposons exhibit a higher integration preference for transcriptional units, they may potentially lead to the activation of oncogenes or the disruption of tumor suppressor genes, thus promoting malignant transformation. It has been reported that no growth advantage was observed in PB-modified primary human cells during a 140-day experiment [152], and no observable tumor formation was found in livers of wild-type mice modified with PB for one year [153]. In addition, transposon integrations can be redirected to a demonstrated safe-harbor site, which can be achieved by transposase modified to carry site-specific DNA binding domains at its N- or C-termini [82]
- Investigators have found that the probability of plasmid backbone DNA integration is relatively high in PB-modified human embryonic kidney (HEK-293) cells [152]. Although this problem can be nearly eliminated by flow cytometry to sort cells based on characteristic elements in the integrated backbone, it still requires close attention. Besides, it remains to be further explored if plasmid backbone integration exists in clinically relevant cells and animal models
- Previous studies [51] have reported that the 5′ PBITR has potential promoter activity. To avoid possible influence, gene-trap cassettes could be placed opposite the 5′ PBITR for chromatin integration [52]
- Despite the widespread assumption that nonviral vectors should not elicit any immune response, foreign DNA itself has the potential to activate the innate immune system [154]. Thus, some immune regulation may still be needed after stable transposition into the host genome

## 5. Conclusions and Perspectives

Transposon-based technologies hold great promise for the development of powerful genomic tools. There is no doubt that there will be more reports in the future using PB for gene delivery in stem cells and other fields of research. By combining transposon technology with accurate gene editing techniques, the continued development, refinement, and clinical transformation using PB may herald an exciting and promising new era of gene therapy.

## Figures and Tables

**Table 1:** Genetic screening using PB transposon.

	Screening purpose	Cell type	Strain	Genes identified	Reference
*Loss of function*					
Drug resistance	6-thioguanine	*Blm*-deficient ESCs	Mouse	*Dnmt1*	Guo et al., 2004 (ref [96])
	6-thioguanine	haESCs	Mouse	*Msh2, Hprt*	Leeb et al., 2011 (ref [100])
	6-thioguanine	haESCs	Mouse	Mismatch repair genes *(Msh2, Msh6, Mlh1)*	Pettitt et al., 2013 (ref [124])
	Olaparib (PARP inhibitor)	haESCs	Mouse	*Parp1*	Pettitt et al., 2013 (ref [124])
	Talazoparib (PARP inhibitor)	haESCs	Mouse	*Ewsr1*	Pettitt et al., 2017 (ref [113])
	Doxorubicin	haESCs	Mouse	*Rmi2，Pdk4,* *and Acbd6*	Liu et al., 2017 (ref [91])
	Tetrodotoxin-like toxicant	haNPCs	Rhesus monkey	*B4GALT6*	Wang et al., 2018 (ref [38])
	6-thioguanine	haTSCs	Mouse	*Hprt*	Cui et al., 2019 (ref [155])
	Puromycin	haESCs	Mouse	*Lrp6*	Mao et al., 2020 (ref [126])
Stem cell-related characteristics	Exit from self-renewal	haESCs	Mouse	*Zfp706, Pum1*	Leeb et al., 2014 (ref [108])
	Exit-from-pluripotency	haESCs	Mouse	*Garnl3, Ifltd1, Sema5a, Cdk5rap2* and *Phf21a*	Liu et al., 2017 (ref [91])
	Spongiotrophoblast specification	haiTSCs	Mouse	*Htra1*	Peng et al., 2019 (ref [118])
	Reprogramming factors	haEpiSCs	Mouse	*Hs3st3b1*	Gao et al., 2021 (ref [122])
	Haploidy maintenance	haESCs	Mouse	*Etl4*	Zhang et al., 2020 (ref [123])
*Gain of function*					
Drug resistance	PluriSIn-1(SCD1 inhibitor)	ESC	Human	RAS pathway genes	Weissbein et al., [2019] (ref [127])
Stem cell-related characteristics	Ground state pluripotency	EpiSCs	Mouse	*Nr5a*	Guo et al., 2010 (ref [35])
	Cell differentiation	ESC	Human	*RHOA*	Gayle et al., 2015 (ref [156])
	Growth advantage	ESC	Human	RHO-ROCK pathway genes	Weissbein et al., 2019 (ref [127])
	Teratoma formation	ESC	Human	PI3K-AKT and HIPPO pathways genes	Weissbein et al., 2019 (ref [127])

**Table 2:** Comparison of genome-wide screening libraries based on cDNA, RNAi, CRISPR/Cas9, and PB transposons.

	cDNA library	RNAi library	CRISPR/Cas9 library	*piggyBac* library
Work mode	Gain of function	Loss of function	Loss of function/gain of function	Loss of function/gain of function
Vehicle	cDNA	Sh/siRNA	sgRNA	*piggyBac* transposon plasmids
Targeting restrictions	Part of transcripts	Only targets mRNA	Protospacer adjacent motif (PAM) must be present	Only at TTAA site
Mutagenesis efficiency	≥2 standard deviations induced expression signals	≥70% gene knockdown	Knockout, knockdown, or overexpress with different kinds of libraries achievable	Activation levels of genes variable, inactivation achievable in haploidy
Genome coverage	Depend on library design	Depend on library design	Depend on library design	Genome-wide in principle, but influenced by integration site preference
Types of mutations	Overexpression	Knockdown	Chromosomal deletions and translocations	Gene activation and inactivation are due to transposon insertion
Reversibility	Potentially reversible	Potentially reversible	Knockdown or overexpressed libraries reversible, knockout libraries irreversible	Reversible
Limitations	Abundance of transcripts varies	High offtarget effects	Less offtarget effects	Biallelic gene inactivation rare in diploid cells; integrations for activation need to be upstream of the transcription start site
Cytotoxicity	Variable to high	Variable to high	Low	Low

## Data Availability

No data is available.
